# Biphasic modulation of insulin signaling enables highly efficient hematopoietic differentiation from human pluripotent stem cells

**DOI:** 10.1186/s13287-018-0934-x

**Published:** 2018-07-27

**Authors:** Fuyu Duan, Rujin Huang, Fengzhi Zhang, Yonglin Zhu, Lin Wang, Xia Chen, Lufeng Bai, Wei Guo, Sophia Chia-Ning Chang, Xiaoyu Hu, Jie Na

**Affiliations:** 1 Center for Stem Cell Biology and Regenerative Medicine, School of Medicine, Tsinghua University, Beijing, 100084 China; 2 School of Clinical Medicine, Tsinghua University, Beijing, 100084 China; 3 Institute of Immunology and School of Medicine, Tsinghua University, Beijing, 100084 China; 4 Zhejiang University International Campus, Haining, Zhejiang Province China; 5 School of Medicine, China Medical University, Taichung, Taiwan

**Keywords:** Human pluripotent stem cells, Hematopoiesis, Insulin, Serum free

## Abstract

**Background:**

Hematopoietic lineage cells derived from human pluripotent stem cells (hPSCs) hold great promise for the treatment of hematological diseases and providing sufficient cells for immune therapy. However, a simple, cost-effective method to generate large quantities of hematopoietic stem/progenitor cells (HSPCs) is not yet available.

**Methods:**

We established a monolayer, chemically defined culture system to induce hematopoietic differentiation from hPSCs in 8 days.

**Results:**

We found that insulin-free medium allowed hPSCs to leave pluripotency promptly and preferably enter the vascular lineage. Addition of insulin during the later stage of differentiation was essential for the efficient induction of hemogenic endothelium and the emergence of large numbers of CD34^+^CD43^+^ HSPCs, while no insulin condition preferably permits endothelial differentiation. Global transcriptome profiling revealed that HSPCs differentiated using our protocol were similar to embryoid body-derived HSPCs. HSPCs obtained from our differentiation system formed robust erythroid, granulocyte and monocyte/macrophage colonies in CFU assay, and can be induced to generate functional macrophages with robust phagocytic ability.

**Conclusion:**

Our results demonstrated that proper manipulation of insulin-mTOR signaling can greatly facilitate HSPC formation. This finding can be further exploited to formulate cost-effective differentiation medium to generate large quantities of cells of desired blood lineages for regenerative medicine.

**Electronic supplementary material:**

The online version of this article (10.1186/s13287-018-0934-x) contains supplementary material, which is available to authorized users.

## Background

Human pluripotent stem cell (hPSC)-derived hematopoietic stem and progenitor cells (HSPCs) may be used to model human hematopoietic differentiation, to produce large quantities of blood lineage cells for therapeutic purposes and to model human hematological diseases [1, 2]. Several methods of HSPC differentiation have been developed. Some protocols used embryoid bodies (EBs) as a differentiation intermediate, followed by coculture on feeder cells, such as OP9 [3–5]. As the cell types in EBs are highly heterogeneous, the differentiation efficiency is relatively low. Moreover, cultures on mouse feeder cells lead to human cells in contact with cells of foreign species, which may hinder downstream therapeutic applications [6]. Chemically defined differentiation protocols were also developed, but often involve culturing in specialized medium and many cytokines, as well as many steps, which make the cost of HSPCs very expensive and the large-scale production of HSPCs impractical [7, 8].

During embryo development, HSPCs are formed very early in the yolk sac mesoderm from nascent hemogenic endothelial cells [9]. Later, the hematopoietic sites shift to the aorta-gonad-mesonephros (AGM) and fetal liver while the placenta continues to be a site of blood cell formation [9]. To achieve efficient in vitro differentiation, it is important to follow the developmental principle of the desired lineage. Endothelial cells, cardiac cells and HSPCs were all progenies of the lateral mesoderm [10]. It has been well established that insulin withdrawal from the differentiation medium could promote highly efficient mesoderm and cardiomyocyte differentiation [11, 12]. Recently, it was reported that rapamycin can facilitate HSPC formation through enhancing endothelial differentiation [13]. As rapamycin is an inhibitor of the mTOR pathway, which is downstream of insulin signaling [14], we hypothesize that appropriate modulation of insulin can improve HSPC differentiation from hPSCs.

In this study, we aim to dissect the role of insulin/mTOR signaling during the process of hPSC differentiation toward HSPCs and establish a chemically defined and cost-effective system to generate human HSPCs efficiently. We found that insulin withdrawal markedly accelerated vascular mesoderm induction from hPSCs. However, insulin-mTOR signaling is required for hemogenic endothelium development and the formation of HSPCs. By modulating Wnt signaling during the mesoderm induction stage and inhibiting TGF-β signaling after hemogenic endothelium induction, one can trigger highly rapid and efficient HSPC formation in monolayer culture, without feeder cells and hematopoietic cytokines. These HSPCs formed erythroid (CFU-E), granulocyte (CFU-G), macrophage (CFU-M), granulocyte–macrophage (CFU-GM) and mixed myelo-erythroid (CFU-mixed) colonies in in vitro colony-formation assays, and can be induced to become more mature HSPCs by cocktail of hematopoietic cytokines or develop into macrophages by monocyte cytokines. The hPSC-derived macrophages exhibited robust phagocytic ability toward human pathogens. Thus, our system offers a simple, cost-effective platform to produce HSPCs from hPSCs for research and regenerative medicine.

## Methods

### hPSC culture and HSPC differentiation

H1 and hiPSCs were routinely maintained on MEF feeders in the hESC medium: KnockOut DMEM (Gibco) culture medium supplemented with 20% (vol/vol) KnockOut serum replacement (Gibco), 1% nonessential amino acids (Gibco), 1 mM l-GlutaMAX-I (Gibco), 0.1 mM β-mercaptoethanol (Sigma-Aldrich) and 8 ng/ml bFGF. They were passaged with 1 mg/ml collagenase IV (Invitrogen) and seeded onto a 25-cm^2^ flask previously coated with 0.1% gelatin solution (Sigma Aldrich). For feeder-free and serum-free culture, hESCs were maintained on Matrigel (BD Biosciences)-coated plates (Corning) in TeSR-E8 medium (STEMCELL Technologies). The human iPSCs used in our study were reprogrammed from cord blood CD34^+^ cells using the CytoTune™-iPS Reprogramming Kit (Invitrogen) according to the manufacturer’s instructions. The umbilical cord blood was obtained from Beijing 301 Hospital with informed consent from the parents, approved by Tsinghua University research ethics committee. CD34^+^ cells were purified by CD34 magnetic beads (MACS). This iPSC line was fully characterized and used in another two published studies [15, 16]. Both of H1 hESCs and hiPSCs have normal diploid karyotypes (Additional file 1: Figure S1).

For hPSC hematopoietic differentiation, undifferentiated hESCs cultured in TeSR-E8 medium were digested into single-cell suspension by 1 mg/ml accutase (Millipore) and plated onto Matrigel-coated culture dishes at a density of 2 × 10^4^ cells/cm^2^ in TeSR-E8 medium with Y27632 (TOCRIS) (10 μM). After 24 h, Y27632 was withdrawn from the medium and cells were cultured for another 24 h. Next, cells were induced by first culturing in basal medium which is RPMI 1640 medium supplemented with 2% B27 (Gibco, with or without insulin), 1% l-GlutaMAX-I and 50 μg/ml ascorbic acid (Sigma Aldrich). Then 5 ng/ml BMP4 (R&D) was added for 24 h. Afterward, the medium was changed to basal medium containing 5 ng/ml BMP4 and 2 μM GSK3 inhibitor CHIR99021 (TOCRIS) for another 48 h. In differentiation stage II, cells were replated onto Matrigel-coated dishes at a density of 4 × 10^4^ cells/cm^2^ in basal medium with 50 ng/ml VEGF165 and 10 ng ng/ml FGF2 for 48 h. Then, the medium was replaced with basal medium with 50 ng/ml VEGF165, 10 ng ng/ml FGF2 and TGFβ inhibitor SB431542 (10 μM; TargetMol) for another 72 h. Finally, floating cells were collected on differentiation day 8 and transferred into low-attachment plates and cultured in StemSpan medium (STEMCELL Technologies) supplemented with 50 ng/ml SCF, 50 ng/ml Flt3, 50 ng/ml TPO,10 ng ml IL-3 and 20 ng/ml IL-6 (cytokines all from Peprotech) for 1 week.

### RNA isolation, Q-PCR, and high-throughput RNA sequencing

Total RNA was isolated from undifferentiated hPSCs, and the differentiated cells were sorted using the RNeasy Plus Mini Kit (Qiagen) and treated with RNase-free DNase. Then 1 μg RNA of each sample was reverse-transcribed with Superscript III (TransGen). Q-PCR reactions were performed using GoTaq qPCR Master Mix (Promega) in a CFX96 Real-Time System (Bio-Rad) and the results were analyzed with the Bio-Rad CFX Manager program. The sample input was normalized against the critical threshold (Ct) value of *GAPDH* or *ACTB*. Primer sequences are presented in Additional file 2: Table S7. For high-throughput RNA sequencing, cDNA libraries were prepared using the TruSeq™ RNA Sample Preparation kit (Illumina) and sequencing was performed using GENEWIZ (http://genewiz.com.cn). The clean reads were mapped to the human reference genome (hg38) using BWA software. Cluster analysis of gene expression patterns was performed using Cluster 3.0 and JavaTreeview software (http://bonsai.hgc.jp/~mdehoon/software/cluster/software.htm). GO term enrichment was analyzed using DAVID (http://david.abcc.ncifcrf.gov/). Data are publicly available at the National Center for Biotechnology Information with Gene Expression Omnibus (GEO) accession number GSE114159.

### FACS analysis

Cells were dissociated into single cells with 0.05% trypsin (0.1% EDTA) wherever suitable or rinsed off from culture, and resuspended in a FACS washing buffer (PBS with 5% fetal calf serum (FCS) and 2.5 mM EDTA). The cell suspension was then stained with the desired antibodies. The antibodies used in this study were: CD56 (1:50, clone CMSSB; eBioscience), PE-conjugated CD309 (FLK1, 1:50, clone 7D4–6; Biolegend), PE-conjugated CD13(1:50, clone WM15; BD), FITC-conjugated CD31 (1:50, clone WM59; BD), APC-conjugated CD34 (1:50, clone 581; BD), FITC-conjugated CD43 (1:50, clone MEM-59; Biolegend), PE-conjugated CD43 (1:20, clone eBio84-3C1; eBioscience), FITC-conjugated CD45(1:50, clone 5B1; Miltenyi Biotec), FITC-conjugated CD14 (1:50, clone HCD14; Biolegend), PE-conjugated CD68 (1:50, clone Y1/82A; Biolegend), APC-conjugated CD11b (1:50, clone ICRF44; Biolegend), PE-conjugated CD163 (1:50, clone RM3/1; Biolegend), PE-conjugated CD73 (1:50, clone AD2; Biolegend) and APC/Cy7-conjugated CD163 (1:50, clone 12G5; Biolegend). FITC-conjugated mouse IgG2a (1:20, 130-098-846; Miltenyi Biotec), APC-conjugated mouse IgG1 (1:20, 130-098-877; Miltenyi Biotec) and PE-conjugated mouse IgG1κ (1:20, clone P3.6.2.8.1; eBioscience) were used as isotype-matched negative controls. Data were collected with a FACS Calibur flow cytometer (BD) and analyzed using FlowJo software, version 10.0.7.

### Immunostaining analysis

Cells were fixed with 4% paraformaldehyde, permeabilized in 0.5% Triton X-100 (Sigma), blocked in 10% normal goat serum (Origene) and then incubated with primary antibodies against CD31 (1:100, clone 2F7B2; Santa Cruz), CD34 (1:100, clone 581; BD) and VE-cadherin (1:100, #2158; Cell Signaling) at 4 °C overnight and detected by DyLight 488 or 549-conjugated secondary antibodies (Thermo). Nuclei were stained with DAPI (Sigma). A Nikon TiE fluorescence microscope was used for image acquisition.

### CFU assay

Day 8 CD34^+^CD43^+^ cells of indicated numbers in 100 μl RPMI 1640 with 2% B27 supplement were mixed with 2 ml MethoCult GF^+^ 4435 (STEMCELL Technologies). The mixture was transferred into one well of a 12-well plate (Corning). The cells were incubated at 37 °C in 5% CO_2_ for 14 days, and the colonies were counted. Each type of colony was classified on the basis of morphological characteristics. Images were taken by Olympus microscope with a DP72 camera.

### Macrophage differentiation

Day 8 CD34^+^CD43^+^ cells were culture in RPMI 1640 supplemented with 2% B27, 1% penicillin–streptomycin (Life Technologies), 25 ng/ml human IL-3 and 50 ng/ml human M-CSF (Peprotech) for the first 7 days. From day 8 onward, the medium was changed to RPMI 1640 with 10% fetal calf serum, 2 mM glutamine, 1% penicillin–streptomycin and 50 ng/ml human M-CSF for 7–10 days.

### Phagocytosis assay

To test the phagocytosis potential of induced macrophages, cells were cultured overnight in 24-well plates at a density of 1 × 10^5^ /ml in RPMI 1640 medium supplemented with 1% penicillin–streptomycin, 2 mM GlutaMAX, 10% fetal calf serum and 50 ng/ml M-CSF. The next day, microorganisms were added at following ratios: cell:GFP-*C. albicans* = 1:10, cell:*M. bovis-BCG* = 1:5 and cell:*M. abscessus* = 1:5. Macrophages were incubated with or without GFP-*C. albicans* for 2 h, or *M. bovis-BCG* and *M. abscessus* for 4 h. Undifferentiated hESCs and CB-MSCs were used as negative controls. After gently washing with PBS three times, cells were analyzed by flow cytometry.

### Statistical analysis

Quantitative data are expressed as mean ± SEM. The statistical significance was determined using Student’s *t* test (two-tail) for two groups or one-way ANOVA for multiple groups. *P* < 0.05 was considered statistically significant.

## Results

### Insulin-free condition promoted highly efficient vascular mesoderm induction

**Fig. 1:**
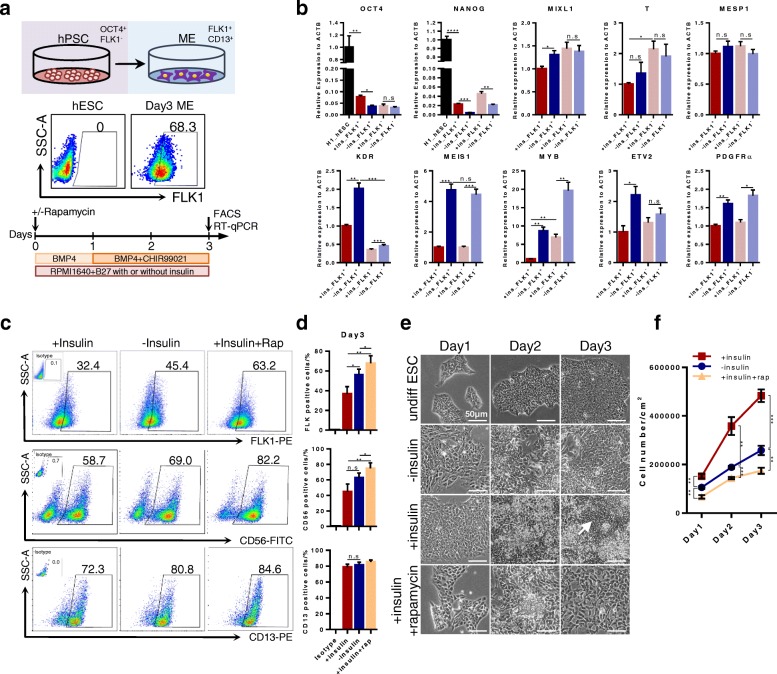
Insulin withdrawal promotes vascular mesoderm differentiation. **a** Schematic view of monolayer-based mesoderm differentiation from hPSCs. Representative flow cytometry results showing proportion of FLK1^+^ cells at differentiating day 3. **b** RT-qPCR analysis of pluripotency (*OCT4*, *NANOG*), mesendoderm (*T*, *MIXL1*), mesoderm (*MESP1*, *KDR*, *MEIS1*, *MYB*, *PDGFRa*) and vascular progenitor cell genes (*ETV2*) at differentiating day 3. *N* = 3 independent replicates. Error bars represent mean ± SEM. n.s. not significant, *P < 0.05, **P < 0.01, ***P < 0.001, ****P < 0.0001. **c** Representative flow cytometry results of vascular progenitor marker FLK1, pan-mesoderm surface marker CD56 and CD13 at differentiating day 3. **d** Statistical analysis of flow cytometry results at differentiating day 3. *N* = 3 independent replicates. Error bars represent mean ± SEM. n.s. not significant, *P < 0.05, **P < 0.01. **e** Representative morphology of differentiating cells from day 1 to day 3. Scale bars, 50 μm. **f** Growth kinetics of differentiating cells under different conditions as indicated from day 1 to day 3. *N* = 3 independent replicates. Error bars represent mean ± SEM. **P < 0.01 ***P < 0.001. FACS fluorescence-activated cell sorting, hESC human embryonic stem cell, hPSC human pluripotent stem cell, ME mesoderm, Rap rapamycin, RT-qPCR reverse transcriptase quantitative PCR

It has been reported that insulin has an inhibitory effect toward cardiac differentiation of hPSCs [11], and insulin withdrawal greatly enhanced cardiomyocyte differentiation [12, 17]. Cardiomyocytes, endothelial cells and hematopoietic cells are all derived from mesoderm [10, 18], so we reasoned that insulin withdrawal might also improve endothelial and hematopoietic lineage differentiation. We checked the influence of insulin withdrawal during mesoderm differentiation (Fig. 1a). Cells were cultured in B27-supplemented medium with or without insulin in the presence of BMP4 for 3 days. GSK3 inhibitor CHIR99021 (CHIR) was added for the last 2 days (Fig. 1a). A study recently reported that mTOR inhibition could enhance mesoderm induction [13]. mTORC1 and mTORC2 are also downstream of the insulin-mTOR signaling pathway [14], so we compared the difference of rapamycin, an mTORC1 inhibitor, with insulin withdrawal treatment during mesoderm induction (Fig. 1c, d). The insulin-free condition significantly augmented the FLK1^+^ (36.6 ± 7.6% vs 56.3 ± 5.5%) population compared to the insulin condition (Fig. 1c, d). However, the presence or absence of insulin did not cause significant difference in the percentage of pan-mesoderm markers CD56 (50.0 ± 17.3% vs 62.7 ± 10.8%) and CD13 (78.7 ± 6.5% vs 81.3 ± 6.1%) (Fig. 1c, d), indicating insulin withdrawal specifically promoted vascular mesoderm differentiation. Indeed, we noticed that rapamycin treatment could significant increase both FLK1^+^and CD56^+^ populations in the presence of insulin, which was in accordance with the previous report [13].

We next sorted FLK1^+^ and FLK1^−^ populations at day 3 and analyzed related gene expression. Insulin withdrawal significantly downregulated the expression levels of pluripotent genes *OCT4* and *NANOG*, indicating that cells in this condition were in a more advanced differentiation state (Fig. 1b). In the meantime, the insulin-free condition significantly upregulated mesendoderm genes *MIXL1* in the FLK1^+^ population, but not in the FLK1^−^ population (Fig. 1b). Mesoderm and vascular-related genes *KDR*, *MEIS1*, *MYB*, *ETV2* and *PDGFRA* were also highly expressed in the insulin-free condition both in the FLK1^+^ and FLK1^−^ populations compared to the insulin group (Fig. 1b), implying insulin withdrawal widely activated vascular mesoderm gene expression. Next, we investigated whether the insulin-free condition would affect cell growth (Fig. 1e, f). The cell morphology changed rapidly in both the insulin withdrawal and rapamycin groups while there were still several undifferentiated regions in the insulin group at day 3 (Fig. 1e, arrow), implying a slower differentiation kinetics of cells in insulin-containing medium, which was in agreement with our RT-qPCR results (Fig. 1b). We compared cell growth kinetics among different conditions (Fig. 1f). On differentiation day 3, in the insulin condition the cell number increased from 1.0 × 10^5^ cells/cm^2^ to nearly 5 × 10^5^ cells/cm^2^. Without insulin, the cell number reached close to 3 × 10^5^ cells/cm^2^. In the presence of insulin, rapamycin treatment severely inhibited cell proliferation and the total cell number was the lowest (less than 2 × 10^5^ cells/cm^2^) (Fig. 1f). Taken together, insulin-free condition promoted highly efficient vascular mesoderm induction at a cost of cell growth.

### Biphasic modulation of insulin signaling promoted highly efficient HSPC differentiation

**Fig. 2:**
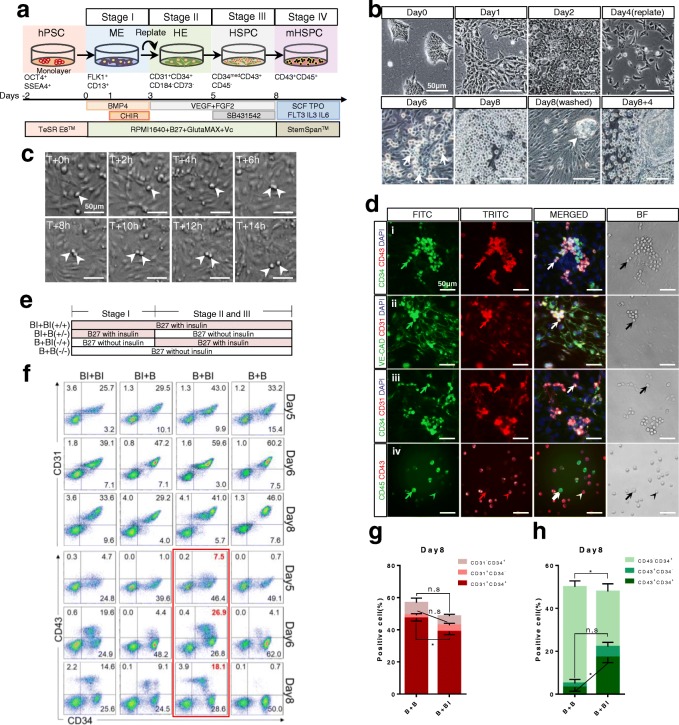
Biphasic modulation of insulin signaling promoted highly efficient HSPC differentiation. **a** Schematic stepwise induction of hematopoietic progenitors from hPSCs. **b** Representative images of stages I–III of hematopoietic differentiation. “Grape-like” clusters at differentiation day 6 indicated by arrows. Scale bars, 50 μm. **c** Time-lapse images of day 6 differentiating cells. Cells undergoing endothelial-to-hematopoietic transition indicated by arrowheads. Scale bars, 50 μm. **d** Representative immunostaining images of day 8 (i–iii) and day 13 (iv) cells for CD34 and VE-cadherin (both green), and CD43, CD31 and CD45 (all red). (i–iii) Grape-like clusters coexpressed CD43, CD34, VE-CAD and CD31(white arrow). (iv) CD45^+^CD43^low^ and CD45^low^CD43^+^ indicated by arrows and arrowheads respectively. Scale bars, 50 μm. **e** Strategy for biphasic insulin treatment. Cells differentiated in B27 without insulin (B) or B27 with insulin (BI). Insulin was added at different stage as indicated. **f** Representative flow cytometry results of surface markers CD31, CD43 and CD34 at day 5, day 6 and day 8. *N* > 3 independent biological replicates. **g** Bar graph of percentage of CD31^+^CD34^+^, CD31^+^CD34^−^ and CD31^−^CD34^+^ cells in B + B and B + BI groups on day 8. *N* > 3 independent biological replicates. **h** Bar graph of percentage of CD43^+^CD34^+^, CD43^+^CD34^−^ and CD43^−^CD34^+^ cells in B + B and B + BI group on day 8. *N* > 3 independent biological replicates. HE hemogenic endothelium, hPSC human pluripotent stem cell, HSPC hematopoietic stem/progenitor cell, ME mesoderm

To investigate whether insulin withdrawal could support HSPC differentiation, we established a monolayer-based HSPC differentiation protocol based on a reported strategy with modifications [19] (Fig. 2a). Firstly, we confirmed that these procedures could support HSPCs in the presence of insulin. During differentiation, cells showed typical mesoderm morphology from days 2 to 3 (Fig. 1e, Fig. 2b). Upon induction by VEGF and FGF2, cells with endothelial morphology emerged from days 5 to 6. Several “grape-like” clusters started to appear at day 6 after adding TGF-β inhibitor SB431542 (Fig. 2b, arrows). At day 8, many floating cells accumulated around the colony-like regions (Fig. 2b, arrowhead). After washing off and collecting the nonadherent cells, round floating cells continuously emerge from the edge of the colony-like region (Fig. 2b, day 8 + 4). To validate that these floating cells underwent endothelial-to-hematopoietic transition (EHT), we next performed time-lapse imaging at day 6 to monitor the course of HSPC formation (Fig. 2c). Presumptive HSPCs (Fig. 2c, arrowheads) divided and acquired the hematopoietic morphology within 14–16 h (Additional file 3: movie S1). Immunostaining of floating cells at day 8 showed that these cells highly expressed both endothelial and hematopoietic lineage markers CD31, VE-cadherin, CD34 and CD43 (Fig. 2d, i–iii), indicating a dual-differentiation potential stage. To further mature the day 8 floating cells toward hematopoietic fate, we sorted the CD43^+^CD34^+^ progenitors and cultured them in StemSpan™ medium (STEMCELL Technology) supplemented with SCF, FLT3, TPO, IL-6 and IL-3. CD45^+^ cells can be observed after 3–5 days of cultivation (Additional file 4: Figure S3c). Interestingly, as the expression level of CD45 increased, the CD43 intensity reduced (Fig. 2d, iv, arrows). Collectively, these results demonstrated that our stepwise-protocol efficiently generated typical hematopoietic progenitors (CD34^+^CD43^+^) within 8 days.

Next, we investigated during which stage of HSPC differentiation insulin was required, the mesoderm induction stage (stage I) or the hemogenic endothelial formation stage (stages II and III) (Fig. 2e). We first examined the efficiency of HSPC and EC formation in different insulin supplement treatment combinations by monitoring surface markers CD31, CD34 and CD43 from day 5 to day 8. Interestingly, we found that insulin withdrawal did not impair EC differentiation; on the contrary, it significantly augmented generation of the CD31^+^CD34^+^ population from day 6 to day 8 (Fig. 2f, g, B + B group). However, withdrawing insulin from the entire process severely inhibited the generation of CD43^+^ hematopoietic population (Fig. 2f, h, B + B group). This suggests that insulin signaling was required for the EHT process. By comparing these combinations, we found that B27 minus insulin followed by B27 supplement combination generated the most CD43^+^CD34^+^ hematopoietic population at day 8 (Fig. 2f, h, B + BI group), indicating that insulin withdrawal at stage I could promote the hematopoietic and endothelial differentiation. Thus, biphasic modulation of insulin signaling promoted highly efficient HSPC differentiation.

### Insulin withdrawal promoted arterial specification and inhibited hemogenic endothelium formation

**Fig. 3:**
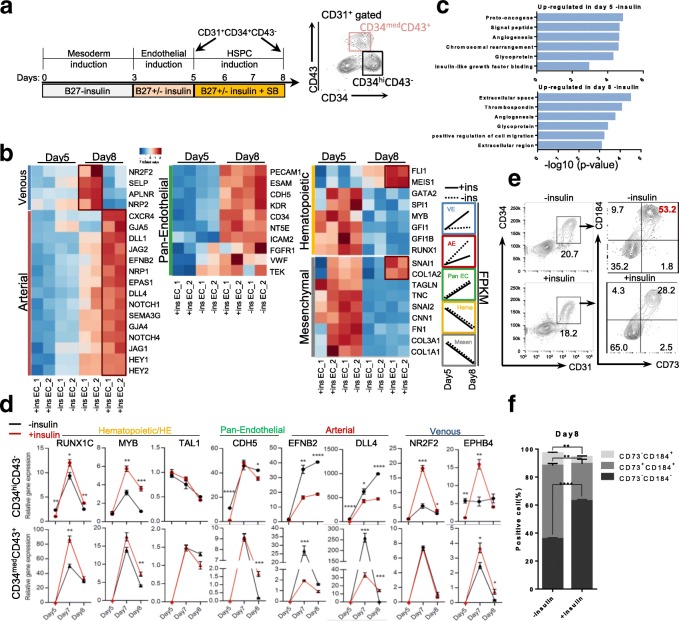
Insulin withdrawal enhanced specification of arterial endothelium and inhibited hemogenic formation. **a** Schematic differentiation protocol and sample collection by flow cytometry. **b** Heatmap analysis of venous (blue), arterial (red), pan-endothelial (green), hematopoietic (yellow) and mesenchymal (gray) genes in day 5 and day 8 sorted CD34^+^CD31^+^CD43^−^ fractions in presence or absence of insulin, respectively. Two replicates performed in each group. **c** GO analysis of top differential upregulated genes in day 5 and day 8 EC fractions in absence of insulin. **d** Representative flow cytometry of day 7 CD184 and CD73 expression in CD31^+^CD34^+^ gated population in presence and absence of insulin, respectively. **e** Bar graph of percentage of CD184 and CD73 expression in CD31^+^CD34^+^ gated population in presence and absence of insulin on day 7, respectively. *N* > 3 independent biological replicates. **f** RT-qPCR analysis of CD34^hi^CD43^−^ and CD34^med^43^+^ subfractions from differentiating day 5 to day 8. HE hemogenic endothelium, HSPC hematopoietic stem/progenitor cell

To find out how insulin signaling affects HSPC emergence, we compared the transcriptome of stage II CD31^+^CD34^+^CD43^−^ hemogenic endothelial progenitor (HEP) cells from plus insulin or minus insulin culture conditions. Day 5 and day 8 CD34^+^CD31^+^CD43^−^ cells in the absence or presence of insulin were sorted by flow cytometry, and then subjected to high-throughput RNA sequencing (Fig. 3a). By day 8, in the insulin-free condition, arterial marker genes (*CXCR4*, *GJA5*, *EFNB2*, *NRP1*, etc.) and pan-endothelial genes (*PECAM1*, *CDH5* and *KDR*) were significantly upregulated (Fig. 3b). In the meantime, venous genes (*NR2F2*, *SELP*, *NRP2*, etc.) were downregulated compared to the plus insulin condition (Fig. 3b). In contrast to endothelial genes, hematopoietic and mesenchymal-related genes decreased at day 8 compared to day 5 (Fig. 3b), implying that EHT had occurred and the remaining ECs gradually took mature endothelial characteristics. GO analysis revealed that angiogenesis and cell adhesion-related genes were enriched in the insulin withdrawal group, indicating a strong endothelial but not hematopoietic signature (Fig. 3c Additional file 5: Table S4 and Additional file 6: Table S2).

We resolved the following two fractions based on CD34 and CD43 expression levels in CD31^+^ gated cells from day 5 to day 8: CD34^hi^CD43^−^ and CD34^med^CD43^+^ (Additional file 7: Figure S2b). In insulin-free conditions, by day 8 the CD34^med/lo^CD43^+^ hematopoietic population only comprised about 10% of cells arising from the CD34^+^CD43^−^ population, which was 2-fold less than the plus insulin condition (10.4% vs 24.4%) (Additional file 7: Figure S2b), implying that the EHT process was greatly weakened by the absence of insulin. Next, we analyzed gene expression dynamics in CD34^hi^43^−^ and CD34^med^CD43^+^ populations by real-time quantitative PCR from day 5 to day 8. The master regulators known for hematopoietic, pan-endothelial, arterial and venous fates showed distinct patterns in these two populations (Fig. 3d). Compared to the insulin-free condition, hematopoietic factors *RUNX1C* and *MYB* in CD34^hi^CD43^−^ were significantly higher in the presence of insulin at day 7 and day 8. Venous factors *NR2F2* and *EPHB4* were also enriched in the plus insulin condition, while arterial related genes such as *EFNB2* and *DLL4* were enriched in the minus insulin condition (Fig. 3d), which was consistent with our RNA-seq results (Fig. 3b).

Arterial endothelial cells and definitive hemogenic endothelial cells can be distinguished on CD73 and CD184 expression [20]. Arterial vascular endothelium (AVE) is characterized as a CD184^+^CD73^med^ population, while hemogenic endothelium (HE) is restricted to the CD73^−^CD184^−^ fraction. We checked the expression pattern of these two markers in CD34^+^CD31^+^ gated cells at day 7. Insulin withdrawal markedly inhibited generation of the CD73^−^CD184^−^ HE population (Fig. 3e, f). Compared with the insulin group, the CD31^+^CD184^+^CD73^+^ population was significantly higher in the insulin withdrawal group, implying a stronger arterial phenotype in the insulin-free condition, which is in accordance with our RNA-seq results (Fig. 3b).

Taken together, these results clearly demonstrated that insulin withdrawal promoted the differentiation of CD31^hi^CD34^hi^CD184^+^CD73^+^ arterial endothelium from CD34^med^CD31^med^ vascular progenitors, while at the same time it repressed the emergence of CD31^med^CD34^med^CD184^−^CD73^−^ hemogenic endothelium, resulting in low output of CD43^+^ HSPCs. Thus, insulin supplement is essential for the formation of HE prior to HSPC emergence.

### HSPCs generated through biphasic modulation insulin signaling showed a transcription profile similar to EB-derived HSPCs and, to lesser extent, in-vivo developed HSCs

**Fig. 4:**
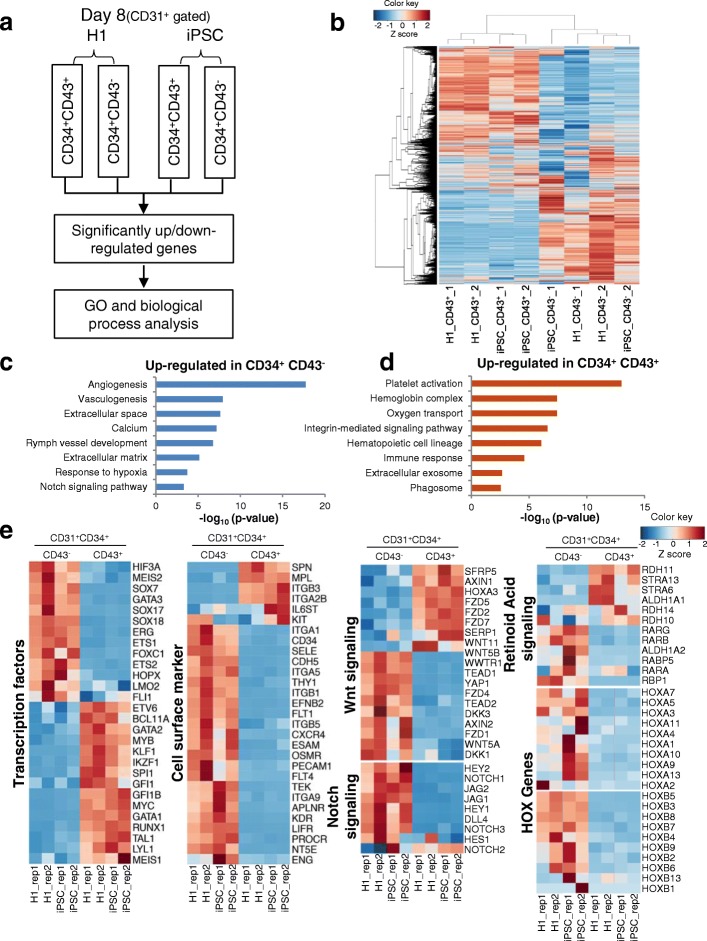
Global transcriptome analysis during HSPC differentiation**. a** Flow cytometry sorting strategy for day 8 H1 and iPSC-derived CD34^+^CD43^−^ and CD34^+^CD43^−^ populations. **b** Heat maps of global transcriptome for H1 and iPSC-derived CD34^+^CD43^+^ and CD34^+^CD43^−^ populations. **c** Upregulated genes enriched in CD34^+^CD43^−^ population. **d** Upregulated genes enriched in CD34^+^CD43^+^ cells. **e** Heat maps of lineage specific transcription factors, surface markers, WNT, NOTCH, RA signaling pathways genes and HOX genes in CD34^+^CD43^+^ and CD34^+^CD43^−^ populations. GO Gene Ontology, iPSC induced pluripotent stem cell

We next analyzed the transcriptome of CD31^+^CD34^+^CD43^−^ and CD31^+^CD34^+^CD43^+^ cells during hPSC to hematopoietic progenitor cell differentiation (Fig. 4a). CD31^+^CD34^+^CD43^−^ and CD31^+^CD34^+^CD43^+^ cells were sorted on day 8 by flow cytometry and analyzed by high-throughput sequencing. CD43^−^ and CD43^+^ cells had very different transcriptomes according to the heatmap and cluster analysis (Fig. 4b). Gene Ontology (GO) analysis revealed that genes highly expressed in CD31^+^CD34^+^CD43^−^ cells were predominantly related to angiogenesis and vasculogenesis, and Notch signaling pathway genes were more highly represented than other developmentally significant signaling pathways (Fig. 4c, Additional file 8: Table S3 and Additional file 9: Table S5). These results suggested that the CD31^+^CD34^+^CD43^−^ cells were mostly ECs. In contrast, CD31^+^CD34^+^CD43^+^ cells highly expressed hematopoiesis-related genes, which are involved in the biological process of platelet activation, oxygen transport and immune response (Fig. 4c, Additional file 8: Table S3 and Additional file 9: Table S5). This implied that the day 8 CD43^+^ cells we obtained resembled the primitive hematopoietic cells during embryo development. Genes of the integrin-mediated signaling pathway were enriched in the CD43^+^ group, reflecting that cells which had gone through the EHT process were more motile hematopoietic cell types (Fig. 4d and Additional file 9: Table S5). We also analyzed in detail the expression pattern of key factors known to participate in hemogenic endothelial formation and hematopoietic system development, the results presented in heatmaps. The transcription factors such as *HIF3A*, *MEIS2*, *SOX17*, *GATA3*, *ETS1*, *ETS2* and *FOXC1* were enriched in the CD43^−^ population, all of them have been shown to play important roles during endothelial development (Fig. 4e), whereas critical genes for hematopoietic development such as *TAL1*, *MYB*, *GATA2*, *RUNX1*, *GFI1B* and *BCL11A* were enriched in the CD43^+^ population (Fig. 4e). Hematopoietic surface marker genes such as *SPN* (CD43), *ITGA2B* (CD41), *KIT* and so forth were highly expressed in CD43^+^ cells. Among them, *ITG3B* (CD61) had been shown to be a marker for hemogenic endothelial cells (HECs) in a recent report [21]. Our results suggested that *ITG3B* could also be a marker for nascent hematopoietic cells just completing EHT. EC specific surface proteins VE-CADHERIN (*CDH5*), TIE2 (*TEK*) and CD73 (*NT5E*) were enriched in CD43^−^ cells (Fig. 4e). Interestingly, CD34, a marker for adult human hematopoietic stem cells, was higher expressed by CD43^−^ cells. This was in agreement with our flow cytometry results that in CD31^+^CD43^−^ cells the CD34 level was high, while in CD31^+^CD43^+^ cells the CD34 level was intermediate (Fig. 3c, Fig. 4e). Notch signaling factors were uniformly enriched in the CD43^−^ population except for *NOTCH2* (Fig. 4e). Both CD43^+^ and CD43^−^ cells expressed a number of WNT pathway genes, and two noncanonical Wnt ligands, *WNT5B* and *WNT11*, were upregulated in CD43^+^ cells, suggesting that the noncanonical Wnt pathway may play a role during HSPC emergence (Fig. 4e). Similarly, genes in the retinoic acid (RA) pathway were found in both CD43^+^ and CD43^−^ cells. Genes involved in receiving the RA signal or metabolizing retinal such as *ALDH1A1*, *RDH11*, *STRA6* and *STRA13* were more abundant in CD43^+^ cells (Fig. 4e)*.*

**Fig. 5:**
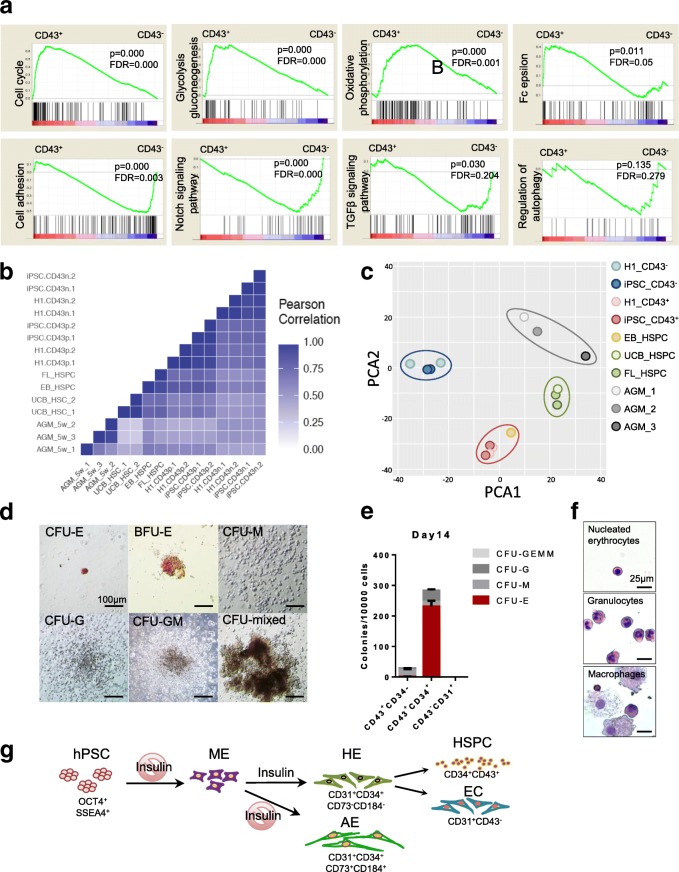
Bioinformatics analysis and characterization of differentiated CD34^+^CD43^−^ and CD34^+^CD43^+^ cells. **a** GSEA enrichment plot of KEGG signaling pathways in H1 hESC-derived CD43^+^ and CD43^−^ populations. Nominal *P* value, empirical phenotype-based permutation test (*P* < 0.05, FDR < 0.25). **b** Pearson correlation map of RNA-seq data of hematopoietic and endothelial-related factors from indicated populations in current study and published datasets. **c** PCA graph of CD34^+^CD43^+^ and CD34^+^CD43^−^ cells derived from H1 hESCs and iPSCs and six kinds of cell types reported in related papers. **d** Colony-forming assay of day 8 CD43^+^CD34^+^ cells. Scale bars, 100 μm. **e** Frequency and percentage of different hematopoietic colonies after HSPCs replated into MethoCult medium for 2 weeks. **f** Wright–Giemsa staining of CD43^+^CD34^+^ cell-derived hematopoietic colonies. Scale bars, 25 μm. **g** Working model for biphasic modulation of insulin in regulating generation of hemogenic endothelium and hematopoietic progenitor cells. AE arterial endothelium, AGM aorta-gonad-mesonephros, CFU colony-forming unit, BFU burst-forming units, E erythroid, EB embryoid body, EC endothelial cell, FL fetal liver, M macrophage, G granulocyte, GM granulocyte–macrophage, HE hemogenic endothelium, hPSC human pluripotent stem cell, HSPC hematopoietic stem/progenitor cell, iPSC induced pluripotent stem cell, ME mesoderm cell, mixed myeloid–erythroid, PCA principal component analysis, TGFβ transforming growth factor beta, UCB umbilical cord blood, FDR false discovery rate

Gene set enrichment analysis (GSEA) coupled with the pathway gene set data of Kyoto Encyclopedia of Genes and Genomes (KEGG) was applied to explore signaling pathways involved in CD34^+^CD43^+^ cell generation. Notably, compared with CD43^−^ cells, the cell cycle, glycolysis and oxidative phosphorylation pathways were all overrepresented in the CD43^+^ population, indicating that mitochondrial aerobic respiration was activated in these cells (Fig. 5a). Cell adhesion, Notch and TGFβ pathways were enriched in the CD34^+^CD43^−^ population (Fig. 5a), which was in accordance with their endothelial characteristics and the hematopoietic induction strategy we used.

To evaluate the developmental stage of our day 8 CD43^+^ cells, we compared their transcriptome with published HSC RNA-seq datasets including CD34^+^ HSCs isolated from the aorta-gonad-mesonephros (AGM) region, fetal liver (FL) and umbilical cord blood (UCB), as well as HSPCs derived through the embryoid body (EB) method [22–24]. Pearson correlation analysis showed that our day 8 CD43^+^ cells resembled the EB-derived HSPCs and FL HSCs the most, suggesting that HSPCs generated using our monolayer differentiation protocol were comparable to HSPCs differentiated from EBs and early embryonic HSCs (Fig. 5b and Additional file 10: Table S6). This was confirmed by the principal component analysis (PCA). CD43^+^ cells derived from H1 and iPSCs were clustered together and close to EB-derived HSPCs (Fig. 5c and Additional file 10: Table S6).

Finally, to test the functionality of the CD43^+^ HSPCs generated after 8 days of monolayer differentiation, we performed a colony-formation assay. CD43^+^CD34^+^ cells were sorted by flow cytometry and plated in MethoCult medium with recombinant cytokines for human HSCs (STEMCELL Technologies). After 14 days, we counted and classified the colonies based on their morphology. Day 8 CD43^+^CD34^+^ cells formed typical erythroid (CFU-E), granulocyte (CFU-G), macrophage (CFU-M), granulocyte–macrophage (CFU-GM) and mixed myelo-erythroid (CFU-mixed) colonies, while CD43^+^CD34^−^ cells had poor ability to generate CFU-E (Fig. 5d, e). In contrast, CD43^−^CD31^+^ cells had no colony-formation ability (Fig. 5e). Wright–Giemsa staining of HSPC-derived hematopoietic colonies had typical morphology of nucleated erythrocytes, granulocyte and macrophage (Fig. 5f), further confirming the hematopoietic identities of CD43^+^CD34^+^ cell-derived clones.

We summarized our findings as a working model depicted in Fig. 5g. Insulin is not required for induction of mesoderm cells from pluripotent stem cells, but is essential for the formation of hemogenic endothelium, which can give rise to both hematopoietic and endothelial lineages. Insulin withdrawal during the mesoderm to vascular induction window will selectively augment the arterial endothelium formation and impair the generation of hemogenic endothelium, subsequently attenuating the hematopoietic potential.

### HSPCs derived from monolayer culture can generate functional macrophages efficiently

**Fig. 6:**
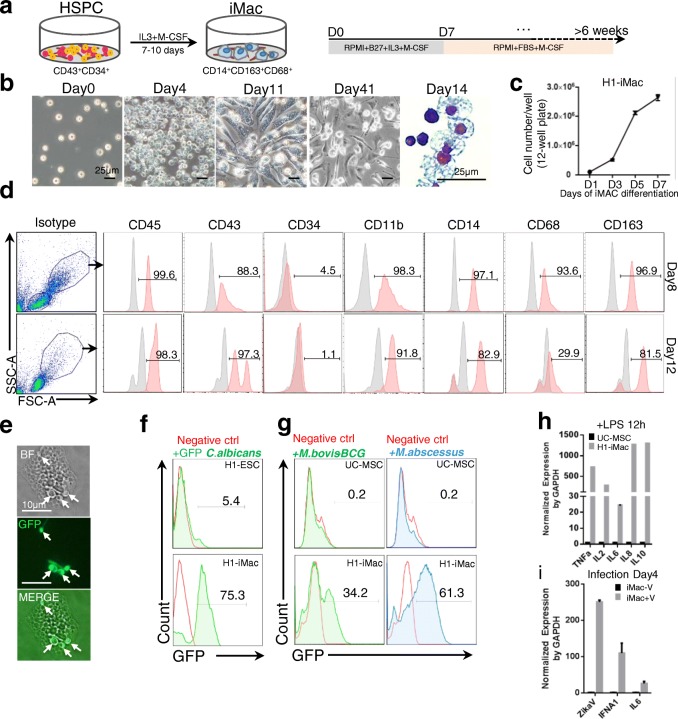
Characterization of HSPC-derived macrophages in vitro**. a** Schematic strategy for generating macrophages from HSPCs. **b** Morphology change during macrophage differentiation assessed by light microscopy and Giemsa staining. Scale bars, 25 μm. **c** Growth kinetics of differentiating iMac in 1 week (*n* = 3 independent experiments, mean ± SEM). **d** Surface marker expression of CD45, CD43, CD34, CD11b, CD14, CD68 and CD163 on H1-Mac analyzed by flow cytometry. **e** Phagocytosis test of differentiated macrophages. Arrows point to several GFP-*C. albicans* engulfed by a macrophage. Scale bars, 10 μm. **f** Flow cytometry analysis of cells’ ability to engulf GFP-*C. albicans*. Control cells: undifferentiated H1 ESCs. **g** Flow cytometry analysis of cells’ ability to engulf GFP-labeled *M. bovis-BCG* and *M. abscessus.* Negative control cells: UC-MSCs. **h** Cytokine gene expression of UC-MSCs and H1-Mac upon stimulation by LPS (mean ± SEM, *n* = 3). **i** Zika viral antigen and cytokine expression in iMac 4 days after virus infection (mean ± SEM, *n* = 3). ESC embryonic stem cell, GAPDH glyceraldehyde 3-phosphate dehydrogenase, HSPC hematopoietic stem progenitor cell, IL interleukin, iMac induced macrophages, LPS lipopolysaccharide, M-CSF macrophage colony-stimulating factor, TNFa tumor necrosis factor alpha, UC-MSC umbilical cord mesenchymal stem cell, BF bright field, GFP green fluorescence protein

A careful examination of colony morphology and Wright–Giemsa staining images identified many macrophages like colonies and cells, suggesting that the primitive HSPCs obtained in our differentiation system may have bias to differentiate toward macrophages. To test this, we sorted CD43^+^CD34^+^ cells and used IL-3/M-CSF to induce HSPCs to form macrophages (Fig. 6a). Remarkably, upon IL-3/M-CSF treatment, after 4 days, the appearance of cells enlarged significantly compared to that at day 0 (Fig. 6b). Typical macrophage morphology could be observed after 10–14 days of differentiation with Giemsa staining (Fig. 6b). The induced macrophages proliferated robustly during the first week, and the cell number multiplied from 1 × 10^4^ cells/well on day 1 to 2.6 × 10^6^ cells/well on day 7, a more than 250-fold increment (Fig. 6c). Flow cytometry analysis of surface markers revealed that most of the macrophages were positive for CD45, CD14, CD11b, CD68 and CD163, but negative for CD34 at day 8 (Fig. 6d, upper panel). Interestingly, we found that CD68 decreased during differentiation at day 12 compared to day 8 (Fig. 6d, lower panel), which resembles the monocyte polarization from M1 (CD68^+^) to M2 (CD163^+^) macrophages in vivo [25, 26].

Next, we investigated whether HSPC macrophages had the ability to phagocytose pathogenic microorganisms. We chose GFP-labeled *C. albicans, M. bovis-BCG* and *M. abscessus* to test. After 2 h incubation, hPSC-derived macrophages engulfed multiple *C. albicans* as indicated by arrows (Fig. 6e). We used flow cytometry to analyze the phagocytic efficiency. About 75% macrophages were positive for GFP, suggesting that they had engulfed GFP-labeled *C. albicans* (Fig. 6f). HPSC-derived macrophages also phagocytosed GFP-*M. bovis-BCG* (34%) and GFP-*M. abscessus* (61%)*,* two kinds of pathogenic bacillus (Fig. 6g), while umbilical cord mesenchymal stem cells (UC-MSCs) failed to uptake any GFP bacillus. We stimulated macrophages with LPS and tested the proinflammatory cytokine gene expression. Upon LPS treatment, the expression of *TNF-α*, *IL2*, *IL6*, *IL8* and *IL10* increased more than 20–1200-fold compared to that in UC-MSCs which served as the negative control cell line, indicating that these cells were capable of inducing inflammatory response (Fig. 6h). Finally, we tested whether these cells can be infected by live zika virus. Viral specific transcript (ZikaV) was detected in macrophages 4 days after infection. Moreover, IFAN1 and IL-6 mRNA were significantly upregulated by viral infection, indicating activation of interferon response and inflammatory process (Fig. 6i). These results strongly suggested that induced macrophages from our biphasic insulin modulation system function normally and may provide a cell model to study the interaction of human macrophages with pathogenic microorganisms.

## Discussion

In this study, we found that by modulating insulin signaling we can significantly improve the efficiency of HSPC generation from hPSCs. We found that the mesoderm induction step is essential to achieve high efficiency in HSPC production later on. Upon insulin withdrawal, and titrated activation of canonical Wnt signaling for 3 days, almost all key regulators of endothelial and early HSPC differentiation were upregulated, such as *MYB*, *MEIS1*, *KDR* and *ETV2* (Fig. 1b). In our system, 1 hESC can generate as many as 1500–2000 CD34^+^CD43^+^ HSPCs after 8 days of differentiation (by estimation). Similar results can be obtained from hiPSCs and other hESC lines. Our protocol is also very simple. We used dissociated single hPSCs cultured in E8 medium as the [27] starting population, followed by mesoderm induction by only BMP4 and CHIR99021 for 3 days. Then cells can be replated as a monolayer for hemogenic endothelium induction followed by HSPC generation. This protocol is cost-effective and may be modified for large-scale production of hemogenic endothelium as well as HSPCs.

### Stage-specific modulation of insulin signaling improves mesoderm and hematopoietic differentiation

Insulin plays a crucial role in glucose and lipid metabolism [27]. Upon activation by insulin, the insulin receptor (IR) tyrosine kinase phosphorylates and recruits different substrate adaptors involved in numerous signaling pathways, such as the PI3K/AKT, Ras/MAPK and PI3K/mTOR pathways, and so on. [27–29]. A previous study screened 400 small-molecule kinase inhibitors and found that the highest enrichment for mesendoderm responders were mTOR inhibitors [13]. In our study, insulin removal during differentiation stage I markedly improved the generation of FLK^+^ cells, indicating that the insulin/PI3K/mTOR pathway negatively regulated vascular mesoderm specification. However, insulin withdrawal may also reduce the activity of the Ras/MAP kinase pathway, which lead to decreased cell proliferation [28]. FGF and VEGF were two cytokines essential for vasculogenesis and angiogenesis, and they both can activate PI3K and MAPK pathways which were required for EC differentiation from PSCs [30]. In our differentiation system, bFGF and VEGF were added during stages II and III, and they may help to maintain the activation level of MAPK and PI3K pathways necessary for EC formation in the absence of insulin. Several ETS family transcription factors (ERG and FLI1) essential for EC formation were downstream of MAPK and PI3K signaling [30–33]. We noticed that without insulin, the EC differentiation efficiency was augmented, but a small percentage of CD34^+^CD43^+^ cells also appeared on day 7 after the addition of TGF-β inhibitor (Fig. 2f, B + B condition). We speculate that insulin signaling is crucial to achieve a high rate of EC to HSPC conversion and subsequent expansion of nascent HSPCs by further stimulating MAPK and PI3K activation and promoting cell proliferation.

### Insulin-mTOR signaling is crucial for HE and HSPC emergence

In the current study, we monitored the EHT process by a monolayer-based hematopoietic differentiation system, which allowed us to evaluate the components that can affect the generation of HSPCs from HE. We investigated the role of insulin during the EHT stage from day 5 to day 8. Similar to a recent report [34], we found that insulin withdrawal during the EHT stage remarkably increased the generation of arterial lineage cells. Furthermore, our results demonstrated that insulin was crucial for emergence of HE and HSPCs (Fig. 2f, Fig. 3e). Another study, for example, found that HE and arterial vascular endothelium (AVE) represented separate lineages [20]. HE is restricted to the CD184^−^CD73^−^ fraction of CD34^+^ cells, while AVE is confined to the CD184^+^CD73^med^ subpopulation. Our results revealed that insulin withdrawal after mesoderm formation would significantly decrease the percentage of HE and attenuate the hematopoietic program. Moreover, we found that CD184^−^ HE was restricted in the CD31^med/lo^CD34^med/lo^ population, but CD184^+^ AVE existed in both the CD31^med/lo^CD34^med/lo^ and CD31^hi^CD34^hi^ populations, indicating that the CD34/CD31 threshold will influence the specification of vascular progenitors, and CD31^med/lo^CD34^med/lo^ is a signature for HE.

mTOR consists of two multiprotein complexes, mTOR complex 1 (mTORC1) and mTOR complex 2 (mTORC2) [14], which are regulated by different upstream signals and have different downstream effectors. RAPTOR and RICTOR are key factors that specifically function in mTORC1 and mTORC2, respectively. Tracing hematopoietic stem cell formation at a single cell level revealed that Rictor was indispensable for HSC emergence from ECs, but was required less in later hematopoietic progression during mouse embryo development [35]. Inhibition of mTORC1 by rapamycin at concentration of 0.1–1 μM can enhance the generation of mesendoderm (OCT4^+^SOX2^−^) cells as well as CD34^+^CD144^+^ hemogenic endothelial cells [13]. In our study, the insulin-free condition at the first stage can significantly improve the generation of FLK1^+^ cells, similar to the effect of rapamycin treatment. Although rapamycin can augment the percentage of FLK1^+^ cells, it significantly decreased the cell number, resulting in low yield of total FLK1^+^ cells compared to the insulin withdrawal group. We also found that insulin was essential for CD43^+^ cell emergence (Fig. 2f, h, B + B group). Insulin withdrawal during the EHT stage (day 5–8) will block the generation of CD43^+^ hematopoietic progenitor cells (Fig. 2f, B + B group). In agreement with this, rapamycin treatment during stage III (day 5–8) would significantly decrease the CD43^+^ population (Additional file 7: Figure S2e), indicating that insulin is likely to function through mTOCR1 to regulate the emergence of HSPCs from HE.

### Transcriptome analysis of HSPCs generated from our system

GO and GSEA analysis revealed that cell cycle genes, *CCNB1*, *AURKA* and *A*URKB, as well as oxidative phosphorylation and glycolysis pathways genes were highly expressed in the CD43^+^ population (Additional file 11: Table S1 Additional file 9: Table S5), suggesting that these cells were in an actively proliferative state. Several inflammation-related genes also showed higher expression in the CD43^+^ population (Fig. 4d), which is in accordance with the report that inflammatory signals played important roles during HSC emergence [36, 37].

HOXA family genes have been shown to play important roles in generating definitive hematopoietic stem cells [23, 24, 38]. A previous study found that hESC-derived HSCs had limited proliferative capacity when compared with somatic HSCs which have higher homeobox (HOX) A and HOXB gene expression levels [39]. Interestingly, in our system, HOXA and HOXB genes uniformly decreased in CD34^+^CD43^+^ cells compared with CD43^−^ cells, implying that HOX genes might be downregulated upon EHT during in vitro differentiation. We have performed transplantation of our hPSC-derived HSPCs into irradiated NSG (NOD.Cg-Prkdc II2rg/SzJ) mice and did not detect any reconstitution (data not shown). Considering the transcriptome of our CD43^+^ cells, they are more likely to be primitive CD43^+^ HSPCs, which have low reconstitution ability in vivo [23].

hPSC-derived HSPCs can be induced to form macrophages at high efficiency, and these macrophages may resemble placenta Hofbauer cells to some extent. Compared to the EB method to obtain macrophages [40], our monolayer method also produced a high number of macrophages and permitted stepwise optimization and analysis of the process. These macrophages highly expressed typical macrophage surface markers, displayed strong phagocytosis activities toward microorganism pathogens and upregulated the expression of proinflammatory cytokines. These hPSC-derived macrophages have great potential to be used as cell models to study the interaction of human specific pathogen with target human cells. Recently, tissue residential macrophages have been shown to play important roles in modulating inflammation and fibrosis response. Disturbance in macrophages will lead to aberrant repair and pathological fibrosis in the heart, lung and liver [41]. hPSC-derived macrophages not only can be a tool to study the progress of fibrosis response, but also a limitless resource for drug screening and therapeutic applications.

## Conclusions

To sum up, we have established a very simple, fast, highly efficient and cost-effective protocol to generate HSPCs and macrophages from hPSCs. We found that insulin withdrawal promoted the rapid exit of hPSCs from pluripotency and enhanced vascular mesoderm differentiation, patterning the endothelial differentiation with an arterial property. However, insulin is vital for emergence and proliferation of nascent hematopoietic progenitors from HE. This protocol can be further modified to screen for small molecules that can regulate the direction of HSPC differentiation, or to scale up to produce large quantities of blood lineage cells for research or the treatment of hematological disorders.

## Additional files

Additional file 1: **Figure S1.** Karyotype confirmation of H1 hESCs and iPSCs used in this study. **a** Normal diploid karyotype of H1 hESCs used in this study. **b** Normal diploid karyotype of CD34 hiPSCs used in this study (PDF 42 kb)

Additional file 2: **Table S7.** Q-PCR primers used in this study (XLSX 11 kb)

Additional file 3: **Movie S1.** Time-lapse movie of HSPC formation on differentiation day 6 (MP4 1.50 MB)

Additional file 4: **Figure S3.** Bioinformatics analysis of human iPSC differentiated ECs and HSPCs. **a** Heatmap analysis of venous, arterial, pan-endothelial, hematopoietic and mesenchymal genes in day 5 and day 8 sorted iPSC-derived CD34^+^CD31^+^CD43^−^ cells in presence or absence of insulin, respectively. Two replicates in each group. **b** GO analysis of top differential upregulated genes in day 5 and day 8 EC fractions in absence of insulin, respectively. **c** Upregulated genes enriched in CD34^+^CD43^−^ population. **d** Upregulated genes enriched in CD34^+^CD43^+^ population. **e.** GSEA enrichment plot of KEGG signaling pathways in H1 hESC-derived CD43^+^ and CD43^−^ populations. Nominal *P* value, empirical phenotype-based permutation test (*P* < 0.05, FDR < 0.25) (PDF 195 kb)

Additional file 5: **Table S4.** GO terms of significantly upregulated genes in day 5 and day 8 HEPs in insulin-free condition (XLSX 268 kb)

Additional file 6: **Table S2.** DEseq2 gene list of significantly differentially expressed genes in day 8 HEPs in presence or absence of insulin (XLSX 303 kb)

Additional file 7: **Figure S2.** Surface marker dynamics during HSPC differentiation. **a** Schematic view of biphasic insulin protocol for HSPC generation. **b** kinetics of CD34 and CD43 expression in CD31 gated cells from day 5 to day 8 in presence or absence of insulin, respectively. **c** Kinetics of CD31, CD34 and CD43 expression from day 3 to day 12. **d** Kinetics of CD34 and CD45 from day 8 to day 19. **e** FACS analysis of CD43 and CD34 expression in presence of insulin and rapamycin. Rapamycin 0.1 μM added from differentiating day 5 to day 8 (PDF 262 kb)

Additional file 8: **Table S3.** DEseq2 gene list of significantly differentially expressed genes in day 8 HSPCs and ECs (XLSX 961 kb)

Additional file 9: **Table S5.** GO terms of significantly upregulated and downregulated genes in day 8 HSPCs versus ECs (XLSX 291 kb)

Additional file 10: **Table S6.** RNA-seq datasets from published papers used for hPSC-derived HSPC comparison (XLSX 33 kb)

Additional file 11: **Table S1.** FPKM value of EC and HSPC global gene expression (XLSX 2681 kb)

## Abbreviations

AGM: Aorta-gonad-mesonephros
CFU: Colony-forming unit
EB: Embryoid body
EC: Endothelial cell
EHT: Endothelial to hematopoietic transition
FACS: Fluorescence-activated cell sorting
FL: Fetal liver
HE: Hemogenic endothelium
HEP: Hemogenic endothelial progenitor
hESC: Human embryonic stem cell
hPSC: Human pluripotent stem cell
HSPC: Hematopoietic stem/progenitor cell
iPSC: Induced pluripotent stem cell
MAC: Macrophage
MEF: Mouse embryonic fibroblast
MSC: Mesenchymal stem cell
UCB: Umbilical cord blood
VE: Venous endothelium
