# Supplementary figures and images for: Biphasic modulation of insulin signaling enables highly efficient hematopoietic differentiation from human pluripotent stem cells
Source: Stem Cell Res Ther. 2018 Jul 27;9:205. doi: 10.1186/s13287-018-0934-x (PMC6062919; doi:10.1186/s13287-018-0934-x)

Figure S1

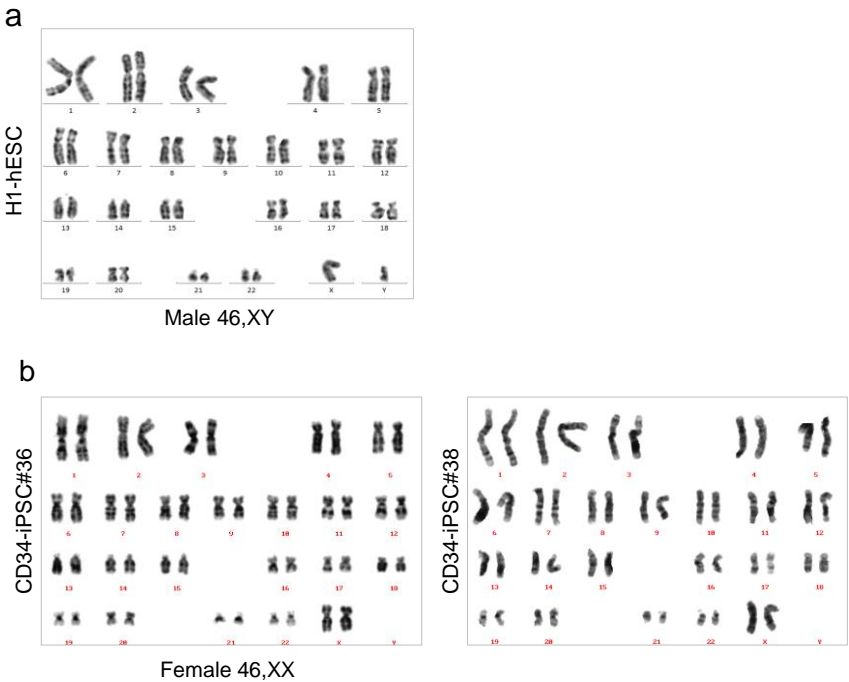

Supplement: Supplementary file 1 — Figure S1. Karyotype confirmation of H1 hESCs and iPSCs used in this study. a Normal diploid karyotype of H1 hESCs used in this study. b Normal diploid karyotype of CD34 hiPSCs used in this study (PDF 42 kb) [file 13287_2018_934_MOESM1_ESM.pdf]

Figure S3

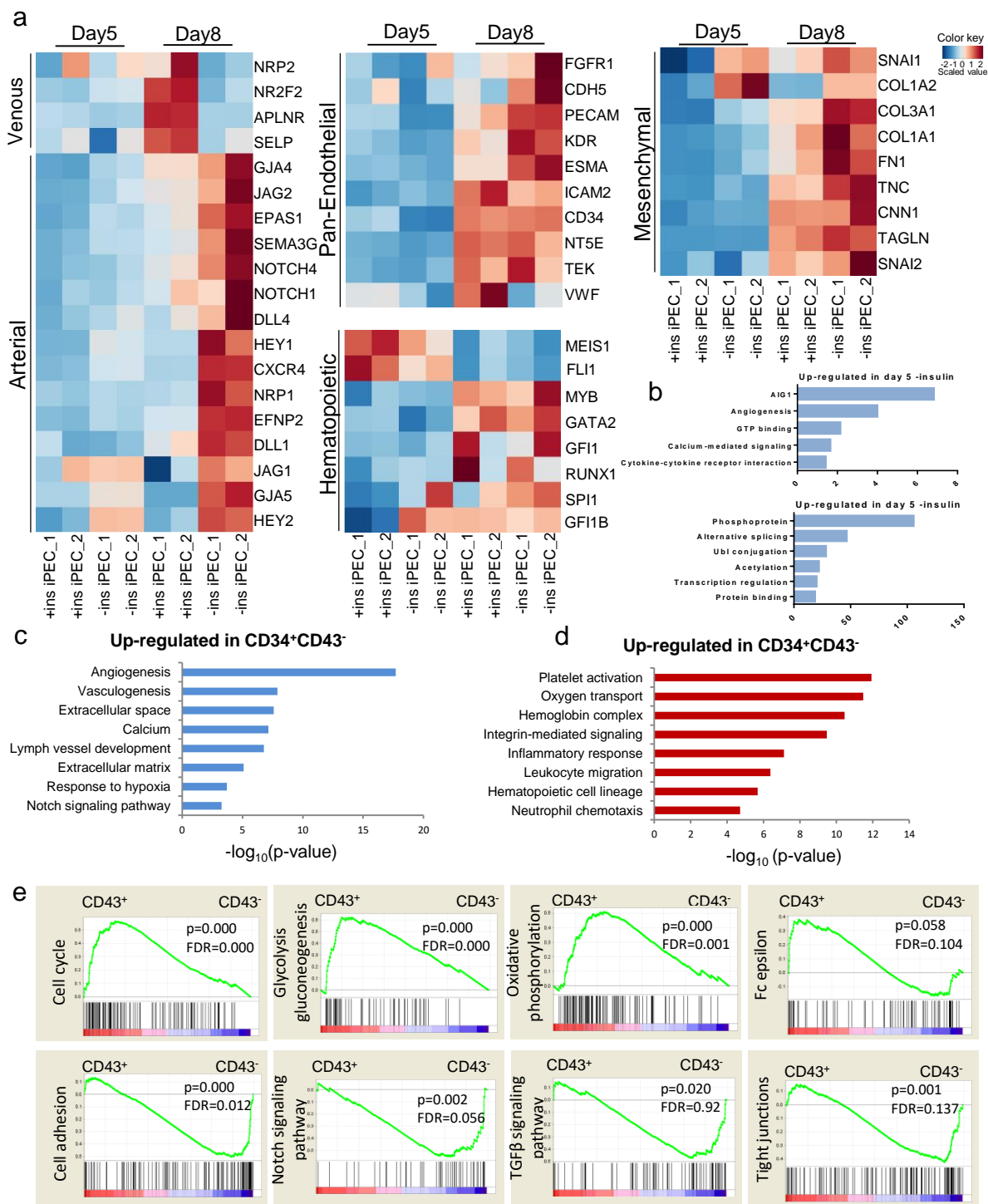

Supplement: Supplementary file 4 — Figure S3. Bioinformatics analysis of human iPSC differentiated ECs and HSPCs. a Heatmap analysis of venous, arterial, pan-endothelial, hematopoietic and mesenchymal genes in day 5 and day 8 sorted iPSC-derived CD34+CD31+CD43− cells in presence or absence of insulin, respectively. Two replicates in each group. b GO analysis of top differential upregulated genes in day 5 and day 8 EC fractions in absence of insulin, respectively. c Upregulated genes enriched in CD34+CD43− population. d Upregulated genes enriched in CD34+CD43+ population. e. GSEA enrichment plot of KEGG signaling pathways in H1 hESC-derived CD43+ and CD43− populations. Nominal P value, empirical phenotype-based permutation test (P < 0.05, FDR < 0.25) (PDF 195 kb) [file 13287_2018_934_MOESM4_ESM.pdf]

Figure S2

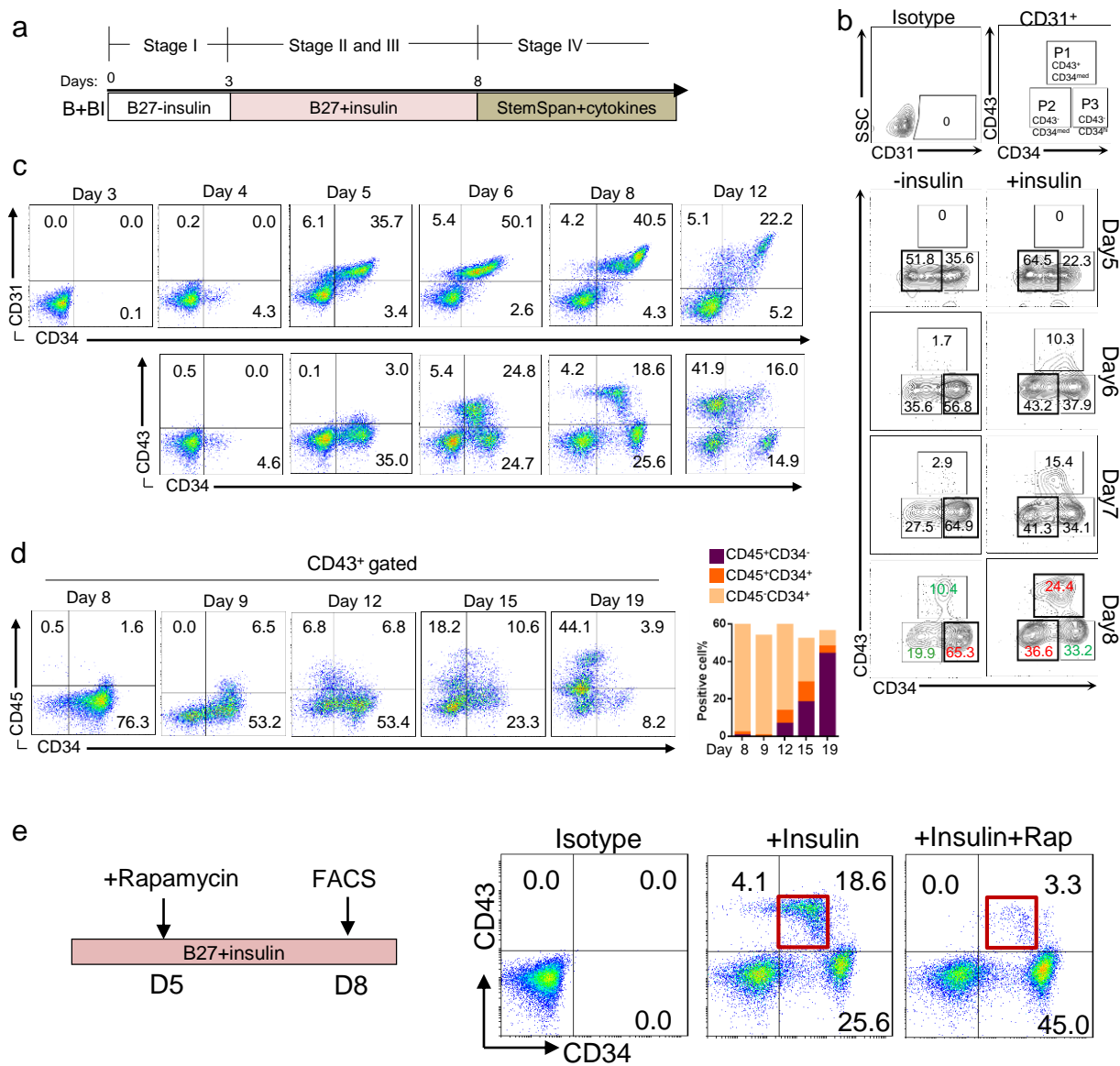

Supplement: Supplementary file 7 — Figure S2. Surface marker dynamics during HSPC differentiation. a Schematic view of biphasic insulin protocol for HSPC generation. b kinetics of CD34 and CD43 expression in CD31 gated cells from day 5 to day 8 in presence or absence of insulin, respectively. c Kinetics of CD31, CD34 and CD43 expression from day 3 to day 12. d Kinetics of CD34 and CD45 from day 8 to day 19. e FACS analysis of CD43 and CD34 expression in presence of insulin and rapamycin. Rapamycin 0.1 μM added from differentiating day 5 to day 8 (PDF 262 kb) [file 13287_2018_934_MOESM7_ESM.pdf]
